# The pharmacology and therapeutic applications of monoclonal antibodies

**DOI:** 10.1002/prp2.535

**Published:** 2019-12-20

**Authors:** María Sofía Castelli, Paul McGonigle, Pamela J. Hornby

**Affiliations:** ^1^ Department of Physiology and Pharmacology College of Medicine Drexel University Philadelphia PA USA; ^2^ Cardiovascular & Metabolic Disease Discovery Janssen R&D LLC Spring House PA USA

**Keywords:** Fc gamma receptors, Fc neonatal receptors, half‐life, pharmacodynamics, pharmacokinetics, protein therapeutic

## Abstract

Monoclonal antibodies (mAbs) have emerged as a major class of therapeutic agents on the market. To date, approximately 80 mAbs have been granted marketing approval. In 2018, 12 new mAbs were approved by the FDA, representing 20% of the total number of approved drugs. The majority of mAb therapeutics are for oncological and immunological/infectious diseases, but these are expanding into other disease areas. Over 100 monoclonal antibodies are in development, and their unique features ensure that these will remain a part of the therapeutic pipeline. Thus, the therapeutic value and the elucidation of their pharmacological properties supporting clinical development of these large molecules are unquestioned. However, their utilization as pharmacological tools in academic laboratories has lagged behind their small molecule counterparts. Early therapeutic mAbs targeted soluble cytokines, but now that mAbs also target membrane‐bound receptors and have increased circulating half‐life, their pharmacology is more complex. The principles of pharmacology have enabled the development of high affinity, potent and selective small molecule therapeutics with reduced off‐target effects and drug‐drug interactions. This review will discuss how the same basic principles can be applied to mAbs, with some important differences. Monoclonal antibodies have several benefits, such as fewer off‐target adverse effects, fewer drug‐drug interactions, higher specificity, and potentially increased efficacy through targeted therapy. Modifications to decrease the immunogenicity and increase the efficacy are described, with examples of optimizing their pharmacokinetic properties and enabling oral bioavailability. Increased awareness of these advances may help to increase their use in exploratory research and further understand and characterize their pharmacological properties.

**Abbreviations**
ADCC: antibody‐dependent cell cytotoxicity
APC: antigen‐presenting cell
CDC: complement‐dependent cytotoxicity
CDR: complementarity‐determining region
CGRP: calcitonin gene‐related peptide
CHO: Chinese hamster ovary cells
CTLA‐4: cytotoxic T lymphocyte‐associated antigen 4
CYP: cytochrome P450
EGFR: epidermal growth factor receptor
Fc: constant fragment
FcRn: neonatal Fc receptor
FcγR: Fc gamma receptor
HAMA: human anti‐murine antibody responses
HEK293: human embryonic kidney 293 cells
IM: intramuscular
IV: intravenous
mAb: monoclonal antibody
pAb: polyclonal antibody
PD‐1: programmed cell death protein 1
PD‐L1: programmed cell death ligand 1
SC: subcutaneous

## 1 INTRODUCTION

It has been said, somewhat facetiously, that pharmacology may be considered a branch of organic chemistry.^1^ In the last century, drugs were made by synthetic chemistry or purified from natural sources (eg, insulin). Pharmacologists developed the principles of drug action in the context of these products to understand their interactions with receptors, transporters, and enzymes (Pharmacodynamics). Similarly, the disposition of drugs within the human body, that is the study of absorption, distribution, metabolism, and excretion (Pharmacokinetics) has been based primarily on small molecules.^2^ Many of these small molecule therapeutics were designed to be highly specific to minimize the undesirable and unpredictable effects of off‐target interactions. However, nature, in the form of the immune system, has developed a sophisticated and extraordinarily effective mechanism for producing long‐lived molecules with highly specific targeting properties. In the last three decades, with the advent of recombinant molecular biology technology and increased understanding of immunological mechanisms, the field has capitalized on these developments, resulting in a dramatic increase in the number of protein‐based therapeutics on the market.

Protein therapeutics with special targeting activity include mAbs and other binding proteins, such as Fc‐Fusion Proteins, according to the classification system proposed by Leader et al.^3^ mAbs are produced by a single clone of B cells, a feature that makes them monospecific and homogeneous.^4^ These characteristics explain their therapeutic potential as compared to polyclonal antibodies (pAbs) produced in vivo. In response to immunization, each B cell expresses antigen region (epitope)‐specific antibodies, leading to slight differences in epitope specificity for each antibody. Thus, pAbs cannot be used therapeutically because, although they have high affinity for the immunizing target, they comprise a mixture of neutralizing and non‐neutralizing antibodies with different affinities. The heterogeneity of pAbs presents problems for their therapeutic characterization due to the different forms of intrinsic activity, making it much more challenging than, for example, a racemic chemical mixture where one stereoisomer is many‐fold more potent than the other.

Antibodies are generated by immunization of animals, with assessment of titers for several months, and then selection of candidate B cells by harvesting spleen cells from the animal. The immune system of the animal generates and optimizes these “lead” molecules. The development of mAbs was made possible after the introduction of the hybridoma technique by Kohler and Milstein in 1975,^5^ a discovery that led to a Nobel Prize. This lymphocyte‐myeloma cell fusion technique generated immortal clones from B cells that could then be screened on the basis of the binding affinity of their product, enabling the selection of specific and high affinity mAbs.^6^ Muromonab‐CD3 (orthoclone OKT3, Janssen‐Cilag) was the first mAb approved for use in humans in 1986. However, since it was of murine origin, patients developed human anti‐murine antibodies (HAMA), resulting in a decrease in the half‐life of muromonab‐CD3 from 18 hours to a few hours, due to increased clearance. In addition, circulating IgE against the mAb led to life‐threatening anaphylactic reactions in response to subsequent treatments.^7^ Since then, genetic engineering has enabled chimeric (mouse/human) mAbs, humanized mAbs by V‐region gene cloning and variable chain complementarity‐determining region (CDR) grafting, as well as fully human mAbs produced by immunization of transgenic rodent models expressing human IgG isotypes.^8^ An alternative to transgenic animals is the use of in vitro libraries, such as phage display, that use a combinatorial screening approach, permitting the selection of moderately high affinity and fully human antibodies.^9^ The resulting mAbs that were discovered by these methods have been developed for a wide variety of immunological, oncological and infectious disease indications.^10^ In the last three decades, approximately 80 mAbs have been granted marketing approval.^11^ In 2018, 12 new mAbs were approved by the FDA, representing 20 percent of the total number of approved drugs. Among these, half of them are expected to reach blockbuster status and generate annual revenues of at least $1 billion by 2024.^12^

Early therapeutic antibodies were targeted to soluble molecules, such as cytokines. However, as the targets of mAbs have broadened to include membrane‐bound targets, the importance of applying pharmacological principles to optimize such therapies has increased. Concepts such as full and partial agonism, biased agonism, and allosteric modulation have become increasing relevant in the characterization of antibody therapeutics. This review will focus specifically on single‐target mAbs, though a similar need exists for pharmacological characterization of antibody‐drug conjugates (ADC), synthetic peptide therapeutics, alternative mAb scaffolds, fusion proteins and bi/multi‐specific mAbs.

## 2 STRUCTURAL AND FUNCTIONAL FEATURES OF ANTIBODIES

Antibodies are large heterodimeric protein molecules (molecular weight ~150 kDa) that consist of two identical light chains and two identical heavy chains, each composed of different domains. The heavy and light chains are held together by disulfide bonds, forming a Y‐shaped structure.^13^ There are five classes of antibodies based on their heavy chain sequences: IgM, IgD, IgG, IgE, and IgA. Each class is divided into different subtypes; for example, IgG is divided into IgG1, IgG2, IgG3, and IgG4. Due to their prolonged circulating half‐life and relative ease of production, all current clinically used therapeutic mAbs are IgGs.^13^

The antigen‐binding fragment (Fab) is made of heavy and light variable chains. The CDR of the variable chains defines the binding site for that mAb (paratope) to the epitope on the antigen.^6^ The paratope is unique to each mAb and is the reason for their target specificity and limited off‐target effects. The crystallizable or constant fragment (Fc) region of mAbs determines their effector function through the ability to bind Fc gamma receptors (FcγR) expressed on endogenous cells.^14^ Binding of mAbs to FcγR on immune cells initiates complement‐dependent cytotoxicity (CDC) and antibody‐dependent cellular cytotoxicity (ADCC). Although Fc effector function is a common feature of antibodies, isotypes IgG1 and IgG3 are the most potent activators of the classical complement pathway.^15^ IgG1 is particularly effective at promoting ADCC. This effector function mediates lysis of the cells bound to IgG1; thus, IgG1 is the most widely used subtype in cancer therapeutic applications, where a cytotoxic effect is desired.^6^, ^16^ On the other hand, IgG2 and IgG4 subtypes have reduced effector function, which can be further diminished by engineering.^17^ These represent a fraction of the currently marketed mAbs 13 but are preferable for immunological indications where ADCC or CDC is not desirable.

## 3 APPROVED INDICATIONS FOR MARKETED mAbs

### 3.1 Immune‐mediated diseases

Monoclonal antibodies have revolutionized the treatment of autoimmune diseases, and several mAbs have been launched in the past three decades for the treatment of these conditions (Table 1). Autoimmune diseases are characterized by the activation of autoreactive CD4^+^ lymphocytes in the peripheral lymph nodes, where naïve T cells interact with antigen‐presenting cells (APCs) and B cells. Activated T cells proliferate and migrate into the disease‐targeted organ parenchyma, where the recognition of endogenous ligands leads to the production of cytokines and pro‐inflammatory molecules, resulting in cell damage and disease progression.^18^ Monoclonal antibodies can target different components of the immune system to suppress the excessive responses that characterize autoimmune diseases.^6^ Some of the mechanisms of mAbs to treat autoimmune disorders include blockade and depletion of T cells and/or B cells, inhibition of the interaction between T cells and antigen‐presenting cells, blockade of T‐ and B‐cell recruitment, blockade of T‐cell differentiation or activation, and blockade of pro‐inflammatory cytokines.^18^ The latter is the most widely used approach, especially the use of mAbs targeting TNF‐α, a cytokine with an essential role in autoimmunity that induces vasodilation and inflammation. These antibodies have been used for the therapy of rheumatoid arthritis for more than a decade, and also show efficacy in psoriatic arthritis, Crohn's disease, ulcerative colitis, psoriasis, and ankylosing spondylitis.

**Table 1:** Therapeutic mAbs used for inhibition of autoimmune reactivity

Antibody	Type	Target	Medical uses
Adalimumab	Human, mAb, IgG1	TNF‐α	Rheumatoid arthritis, Crohn's disease, plaque psoriasis, psoriatic arthritis, ankylosing spondylitis, juvenile idiopathic arthritis
Alemtuzumab	Humanized, mAb, IgG1	CD52	Multiple sclerosis
Belimumab	Human, mAb, IgG1	BAFF	Systemic lupus erythematosus
Benralizumab	Humanized, mAb, IgG1	CD125	Asthma
Brodalumab	Human, mAb, IgG2	IL‐17	Plaque psoriasis
Canakinumab	Human, mAb, IgG1	IL‐1	Cryopyrin‐associated periodic syndrome
Certolizumab pegol	Humanized, Fab’, IgG1	TNF‐α	Crohn's disease, rheumatoid arthritis, axial spondyloarthritis, psoriatic arthritis
Golimumab	Human, mAb, IgG1	TNF‐α	Rheumatoid arthritis, psoriatic arthritis, ankylosing spondylitis
Guselkumab	Human, mAb, IgG1	IL23	Psoriasis
Infliximab	Chimeric, mAb, IgG1	TNF‐α	Rheumatoid arthritis, ankylosing spondylitis, psoriatic arthritis, psoriasis, Crohn's disease, ulcerative colitis
Itolizumab	Humanized, mAb, IgG1	CD6	Psoriasis
Ixekizumab	Humanized, mAb, IgG4	IL‐17A	Plaque psoriasis
Mepolizumab	Humanized, mAb, IgG1	IL‐5	Asthma and white blood cell diseases
Natalizumab	Humanized, mAb, IgG4	Integrin α_ 4_	Multiple sclerosis, Crohn's disease
Ocrelizumab	Humanized, mAb, IgG1	CD20	Rheumatoid arthritis, lupus erythematosus
Omalizumab	Humanized, mAb, IgG1	IgE Fc region	Allergic asthma
Reslizumab	Humanized, mAb, IgG4	IL‐5	Inflammations of the airways, skin and gastrointestinal tract
Risankizumab	Humanized, mAb, IgG1	IL23A	Crohn's disease, psoriasis, psoriatic arthritis, asthma
Rituximab	Chimeric, mAb, IgG1	CD20	Rheumatoid arthritis
Ruplizumab	Humanized, mAb, IgG1	CD154	Rheumatic diseases
Sarilumab	Human, mAb, IgG1	IL6	Rheumatoid arthritis, ankylosing spondylitis
Secukinumab	Human, mAb, IgG1	IL17A	Uveitis, rheumatoid arthritis, psoriasis
Tildrakizumab	Humanized, mAb, IgG1	IL23	Immunologically mediated inflammatory disorders
Tocilizumab	Humanized, mAb, IgG1	IL‐6 receptor	Rheumatoid arthritis
Ustekinumab	Human, mAb, IgG1	IL‐12, IL‐23	Multiple sclerosis, psoriasis, psoriatic arthritis
Vedolizumab	Humanized, mAb, IgG1	Integrin α_4_β_7_	Crohn's disease, ulcerative colitis

### 3.2 Oncology

A number of monoclonal antibodies have been developed for the treatment of various neoplasias, including both hematologic malignancies and solid tumors (Table 2). The first approach is the use of mAbs to target tumor antigens and kill cancer cells.^19^ The main targets for therapeutic mAbs for anticancer indications are growth factor receptors that are overexpressed in tumor cells, such as members of the epidermal growth factor receptor (EGFR) family, including EGFR and HER2.^15^, ^19^ mAbs block these receptors, in turn blocking ligand binding/signaling, which can decrease growth rate, induce apoptosis and sensitize tumors to chemotherapy. An example of this therapeutic approach is the blockade of HER2 receptor by trastuzumab (Herceptin) and other mAbs used for the treatment of HER2‐positive breast cancer. HER2 is overexpressed in 30% of invasive breast cancers.^15^ Trastuzumab inhibits receptor dimerization and internalization, leading to endocytic destruction of the receptor and immune activation.^15^ Other targets besides growth factors include hematopoietic differentiation antigens (CD20, CD30, CD33, CD52), which are glycoproteins found on the surface of normal and tumor cells.^19^ For instance, rituximab (Rituxan), a mAb used for lymphoproliferative disorders, targets CD20, a pan B‐cell marker,^20^ leading to the interactions between FcγR expressed on immune cells and the Fc region of the mAb. FcγR‐dependent activation of immune cells causes the release of inflammatory mediators, directly killing and/or initiating phagocytosis of the opsonized target cells.^21^

**Table 2:** Therapeutic mAbs used for anticancer therapy

Antibody	Type	Target	Medical uses
Alemtuzumab	Humanized, mAb, IgG1	CD52	B‐cell chronic lymphocytic leukemia
Bevacizumab	Human, mAb, IgG2	VEGF	Colorectal cancer, non‐squamous non‐small cell lung cancer, glioblastoma, renal cell carcinoma
Gemtuzumab ozogamicin	Human, ADC, IgG4	CD33	Acute myelogenous leukemia
Trastuzumab‐emtansine	Humanized, ADC, IgG1	HER2	Metastatic breast cancer
Brentuximab‐vedotin	Chimeric, ADC, IgG1	CD30	Hodgkin's lymphoma
Trastuzumab	Humanized, mAb, IgG1	HER2	HER2‐positive breast cancer, gastric/gastroesophageal junction carcinoma
Cetuximab	Chimeric, mAb, IgG1	EGFR	Squamous cell cancer of the head and neck, metastatic EGFR‐positive colorectal cancer
Panitumumab	Human, mAb, IgG2	EGFR	EGFR‐positive metastatic colorectal carcinoma
Ipilimumab	Human, mAb, IgG1	CTLA‐4	Unresectable or metastatic melanoma
Rituximab	Chimeric, mAb, IgG1	CD20	CD20‐positive B cell non‐Hodgkin lymphoma and chronic lymphocytic leukemia
Ofatumumab	Human, mAb, IgG1	CD20	Refractory chronic lymphocytic leukemia
^90^Y‐Ibritumomab Tiuxetan	Murine, mAb, IgG1	CD20	Relapsed or refractory, low‐grade or follicular B‐cell non‐Hodgkin's lymphoma
^131^I‐Tositumomab	Murine, mAb, IgG2	CD20	CD20‐expressing relapsed or refractory low‐grade, follicular or transformed non‐Hodgkin's lymphoma
Atezolizumab	Humanized, mAb, IgG1	PD‐L1	Triple‐negative breast cancer
Avelumab	Human, mAb, IgG1	PD‐L1	Merkel‐cell carcinoma
Blinatumomab	Murine, mAb, IgG1	CD19	Acute lymphoblastic leukemia
Cemiplimab	Human, mAb, IgG1	PD‐1	Metastatic cutaneous squamous cell carcinoma
Daratumumab	Human, mAb, IgG1	CD38	Multiple myeloma
Dinutixumab	Human, mAb, IgG1	GD2	Neuroblastoma
Elotuzumab	Humanized, mAb, IgG1	SLAMF7	Multiple myeloma
Necitumumab	Human, mAb, IgG1	EGFR	Non‐small cell lung cancer
Obinutuzumab	Humanized, mAb, IgG1	CD20	Chronic lymphocytic leukemia
Pembrolizumab	Humanized, mAb, IgG1	PD‐1	Melanoma and other cancers

mAbs can also be used for the selective delivery of radioisotopes selectively to cancer cells. An example is Ibritumab tiuxetan (Zevalin), a mAb labeled with Yttrium 90 or Indium 111, used for the treatment of non‐Hodgkin's lymphoma.^18^ Other mAbs target the tumor microenvironment, with effects such as inhibition of angiogenesis.^15^, ^22^ For instance, bevacizumab (Avastin) blocks the binding of vascular endothelial growth factors, which are overexpressed in various cancers, to the receptor in the vascular endothelium, inhibiting angiogenesis.^15^ Another approach for anticancer mAb‐based therapies is the targeting of immune cells. Also termed immune‐checkpoint inhibitors, these mAbs enhance antitumor immune responses. The main immune‐checkpoint inhibitors target cytotoxic T‐lymphocyte associated antigen 4 (CTLA‐4) and programmed cell death protein 1 (PD‐1)/PD1 ligand 1 (PD‐L1).^23^ CTLA‐4 can be expressed by regulatory T cells infiltrating tumor lesions, and it mediates immunosuppression by inhibiting T‐cell functions. CTLA‐4 blockade restores T‐cell function to kill malignant cells.^24^ Ipilumab (Yervoy), an anti‐CTLA4 mAb, was approved for advanced melanoma in 2011.^25^ The receptor‐ligand pair PD‐1/PD‐L1 negatively regulates T cell‐mediated immune responses and can be used by tumors as a mechanism of evasion of antigen‐specific T‐cell immunologic responses.^26^ Nivolumab (Opdivo), a PD‐1 inhibitor, and atezolizumab (Tencentriq), a PD‐L1 inhibitor, are examples of mAbs that target this immune‐checkpoint pathway, and they have been approved for the treatment of various cancers.^26^ Another immune checkpoint currently being studied for the treatment of cancer is CD40. While CTLA‐4 and PD‐1 are inhibitory immune checkpoints, CD40 is stimulatory. This receptor is a member of the TNF receptor family expressed by B cells and APCs.^15^ Activation of this receptor on APCs leads to the activation of tumor‐specific cytotoxic T cells to eliminate tumor cells. In this case, mAbs in development to target this receptor (eg, dacetuzumab, Seattle Genetics) are agonists, although their clinical efficacy has been limited so far.^27^

### 3.3 Infectious diseases

Table 3 shows mAbs approved for prophylaxis and/or treatment of infectious diseases. The first effective treatment for infectious diseases was the administration of hyperimmune sera from immunized animals or human donors. Although this approach was widely replaced with antibiotic treatment, it still remains useful for the treatment of infectious diseases, including those caused by cytomegalovirus, hepatitis A and B viruses, among others.^28^

**Table 3:** Therapeutic mAbs used for infectious diseases

Antibody	Type	Target	Medical uses
Bezlotoxumab	Human, mAb, IgG1	*Clostridium difficile*	*Clostridium difficile* colitis
Ibalizumab	Humanized, mAb, IgG4	CD4	Multidrug‐resistant HIV infection
Oblitoxaximab	Chimeric, mAb, IgG1	*Bacillus anthracis* anthrax	Anthrax (prophylaxis and treatment)
Palivizumab	Human, mAb, IgG1	F protein of respiratory syncytial virus	Respiratory syncytial virus (prevention)
Raxibacumab	Human, mAb, IgG1	Anthrax toxin	Anthrax (prophylaxis and treatment)
Rmab	Human, mAb, IgG4	Rabies virus G glycoprotein	Post‐exposure prophylaxis of rabies

There are advantages of mAbs for the treatment of infections, over immune sera‐derived preparations, such as low lot‐to‐lot variability, low risk of pathogen transmission, and no immunological complications associated with the use of heterologous sera.^28^ However, the development of mAbs against infectious diseases has been slower in comparison to their development for oncology and immune/inflammatory diseases. The first mAb approved for an infectious disease was palivizumab (Synagis), used for the prevention of severe respiratory disease due to respiratory syncytial virus in high risk populations.^28^ This mAb inhibits virus replication and reduces the frequency of severe disease in premature infants.^28^ Another example is ibalizumab (Trogarzo), approved in 2018 for the treatment of multidrug‐resistant HIV‐1 infection.^29^ This mAb was the first new HIV treatment medication approved in over a decade, and it acts as a post‐attachment inhibitor by binding CD4 receptors and blocking viral entry into the host CD4+ T cells.^30^ Development of other potential mAbs to treat infectious diseases is underway, including Ebola virus disease, hepatitis B and C, herpes simplex virus, among others.^31^

### 3.4 Other indications

Various mAbs have been developed for antiplatelet therapy, although only one, Abciximab, has been approved so far. This is an antibody developed from the murine human chimera c7E3 Fab, which targets the integrin αIIbβ3, preventing integrin binding to fibrinogen and von Willebrand factor, a blood glycoprotein involved in hemostasis.^32^

Another indication for mAbs is the prophylaxis and treatment of migraines. In particular, calcitonin gene‐related peptide (CGRP) is a target for preventative migraine therapy. This peptide acts on the CGRP receptor and is involved in pain modulation, perception, and central sensitization. Since CGRP is elevated in people who suffer from migraines, mAbs targeting this peptide have shown a benefit in these patients.^33^ Examples of this approach include erenumab (Aimovig), fremanezumab (Ajovy), and galcanezumab (Emgality).^34^

There are potential applications of mAbs in the development of immune complex vaccines, as both preventive and therapeutic immunization approaches. Antigen‐mAb immune complex‐based vaccines mimic natural immune complex functions and have been used for poultry for the prevention of infectious bursa disease.^35^ Following this success, several human infectious diseases are being targeted by this approach, including HIV‐1, hepatitis B, and Ebola.^35^

## 4 PHARMACOLOGY OF mAbs COMPARED TO SMALL MOLECULE DRUGS

The many therapeutic applications of mAbs make them desirable to use in experimental pharmacology. However, their use as tool compounds to explore disease pathologies in research laboratories has not kept pace with their clinical utility. This section describes their pharmacology, in comparison to small molecules, to facilitate their use and appropriate experimental design.

Some differences in characteristics and properties between small molecule and mAbs are summarized in Table 4. The most obvious difference is size. Small molecule drugs have a low molecular weight (<700 Da), whereas mAbs are ~150 kDa. The larger size of mAbs limits their potential therapeutic utility to extracellular targets since they cannot access intracellular targets and their distribution to tissue is slower than that of small molecules. Specifically, mAbs do not cross the blood‐brain barrier, and strategies such as intranasal delivery 36 or special targeting 37, 38 must be applied to allow their access into the brain. This limitation can be of benefit where it is desirable to avoid CNS exposure.

**Table 4:** Comparison of the typical pharmacology of small molecules and monoclonal antibodies

Property	Small molecule	Monoclonal antibody
Composition	Synthetic organic compound or natural product	Protein
Mol. Weight	<700 Da	~146 000 Da
Production	Chemical synthesis	Mammalian cells (eg, CHO, HEK293)
Homogeneity	Very homogeneous (>99%)	Heterogeneous, especially glycans
Target affinity	Moderate (nmol/L‐µmol/L)	High (fmol/L‐pmol/L)
Target selectivity	Moderate to High	Very high
Site of action	Binds to nuclear, intracellular or extracellular targets at sites where distributed	Extracellular targets where distributed with very limited CNS exposure
Mode of action	Enzyme activators or inhibitors; receptor agonists (partial, full); antagonists and allosteric modulators	Inhibit or deplete soluble targets and cells (eg, Fc‐mediated ADCC); protein‐protein interactions; agonize (full, allosteric, partial) or antagonize membrane‐bound targets
Multi‐targeting	Dual‐target moderate affinity; polypharmacy low affinity	High affinity bivalent, multivalent including Fc receptors by engineering
Delivery	Oral, occasionally IV, SC, intranasal or inhaled	IV or SC; extremely low oral bioavailability
Absorption and distribution	Entero‐hepatic portal system; capillaries of circulatory system	Lymph and capillaries of blood circulation
Half‐life	4‐24 hours	Weeks
Clearance	Liver, bile or kidney	Intracellular lysosomal degradation
Safety concerns	Usually off‐target; chemical compound related	Antidrug antibodies; target‐related adverse effects; injection site reactions

Typically, small molecules are made by chemical synthesis, leading to homogeneous products of high purity. In addition, the manufacturing process of small molecules is well‐characterized and defined, enabling the rapid production and acceptance of generic versions that are exact copies of the original drug. On the other hand, mAbs are produced by cells, which involves much more variability, such as posttranslational modifications. This can also depend on the cell line used; for example, glycosylation of recombinantly expressed proteins differs between human embryonic kidney 293 cells (HEK293) and Chinese hamster ovary cells (CHO).^39^ Thus, mAbs have a process‐dependent composition consisting of more heterogeneous mixtures. The manufacturing process for mAbs involves cell production of batches with quality control to ensure the product is within predefined parameters. One consequence of this is that in contrast to generic versions of small molecule therapeutics, biosimilar mAbs can only be highly similar to an existing FDA‐approved reference product, with no clinically meaningful differences. In other words, although biosimilars are made with the same recombinant sequence, the resulting product must be clinically characterized to ensure that it is similar enough to the branded mAb.

### 4.1 Pharmacodynamics

The very high affinity and selectivity of mAbs for their molecular target make them less likely to have off‐target effects. This makes them particularly useful as tools to identify the role of a target in disease pathology, especially in experimental models. On the other hand, small molecules may act on several anticipated (and unanticipated) targets and may have compound‐related adverse effects. Similar to small molecules, mAbs may have intrinsic activity as full/partial agonists or allosterically modulate a receptor.^40^ Furthermore, the technology is now available to generate very high affinity bivalent, multivalent, and Fc receptor‐engineered variants.^41^ mAbs can be engineered to have dual targeting efficacy and improve their therapeutic potential by enhancing Fc effector function and improving potency, including ADCC and CDC. An example is obinutuzumab (Gazyva), an anti‐CD20 mAb with enhanced FcγR binding affinity and increased potency compared to the first‐generation antibodies like rituximab.^42^

There are three main methods to generate bispecific mAbs: chemical conjugation with cross‐linkers, somatic fusion of two hybridoma lines, and genetic engineering.^43^ Bispecific mAbs have some advantages over monospecific mAbs, including enhanced cytotoxicity for the treatment of cancer and higher binding specificity by interaction with two different antigens.^43^ They allow for simultaneous binding to cytotoxic T cells and antigen‐expressing tumor cells. This immune‐oncology approach can target cancer cells, for example, catumaxomab (Removab) binds to CD3 on cytotoxic T cells and EpCAM on human adenocarcinomas.^42^ In the last decade, two bispecific antibodies were approved by the FDA for therapeutic use; blinatumomab (Blincyto), used for B‐cell tumors, and catumaxomab, indicated for some liquid tumors 43

mAbs can also be conjugated with highly cytotoxic small molecules (payloads) through chemical linkers, giving rise to ADCs.^44^ ADCs can be used for targeted cancer therapy by conferring selective and sustained cytotoxic drug delivery to tumors, and improving the therapeutic window compared to the use of cytotoxic agents alone.^45^

An example of an ADC is trastuzumab‐emtamsine (Kadcyla ®), a breakthrough formulation that targets the HER2 receptor and delivers emtasine to cancer cells in HER2‐positive metastatic breast cancer.^45^

### 4.2 Pharmacokinetics

Given their large size, polarity, limited membrane permeability and poor gastrointestinal stability, mAbs do not have good oral bioavailability (<<1%).^13^, ^46^ For this reason, they are usually not administered orally, and parenteral administration is mostly by intravenous (IV), subcutaneous (SC), and intramuscular (IM) injections. When mAbs are injected IM or SC, the absorption process from the site of injection is through the interstitial space and into the lymphatic system, with subsequent draining into the systemic circulation.^13^ Although IM and SC routes of administration offer lower bioavailability because of proteolytic degradation in the interstitial fluid or the lymphatic system,^46^ the SC route is the most widely used due to convenience and the possibility of patient self‐administration.^42^ For these last two routes of administration, the peak plasma concentration after a single dose is achieved 3‐7 days after administration, due to the slow absorption into the systemic circulation.^42^, ^46^ Other potential routes of administration include intravitreal, intraperitoneal, and pulmonary.^46^

Compared to small molecules, mAbs have a very long half‐life in circulation, typically 11‐30 days in humans, and thus require much lower dosing frequencies.^42^ The IgG Fc region has a recognition domain for the neonatal Fc Receptor (FcRn), which is constitutively expressed in the vascular endothelium and recycles IgG by receptor‐mediated endocytosis. This protects IgG from lysosomal degradation and allows its trafficking and release back into the circulation, thus increasing its half‐life.^47^ The FcRn trafficking of mAbs and pH‐dependence of this process are discussed in more detail below. Importantly, this mechanism is not easily saturated at therapeutic mAb concentrations,^46^ and has been leveraged to further extend the half‐life of mAbs.^48^

From the site of administration, mAbs extravasate into the tissues and distribute in the interstitial space, followed by binding to tissue components and clearance. Extravasation occurs mainly through convective transport and transcytosis through vascular epithelial cells.^13^ mAbs have low volumes of distribution at steady state (3‐8 L) indicating that they are primarily present in the systemic circulation.^42^ When mAb targets are located in tissues, slow distribution from the systemic circulation may hinder clinical responses, as is demonstrated by the targeting of tumor tissues. A way to overcome this challenge is the use of antibody Fab fragments or single‐chain variable fragments, which can get to the tissue more easily.^46^ Drug‐specific features that affect mAbs distribution to particular tissues include binding affinity to target antigens, target internalization rate and mAb hydrophilicity and charge. Optimization of these factors can be done to improve the distribution of mAbs to target organs.^42^ mAb distribution can be studied using physiologically based pharmacokinetic modeling to describe the process of convection as a product of the lymph flow rate and an efficiency term, as well as integrated analytical tools.^13^ Examples of successful PK/PD modeling where preclinical data can be used to project the human efficacious dose have been reported.^49^

Most small molecule drugs are metabolized through cytochrome P450 (CYP) and transferase enzymes in the liver and excreted through the bile or the kidney. Due to their large size, mAbs cannot be filtered in the kidney and eliminated in the urine, and filtered smaller fragments are typically reabsorbed.^13^ The fact that mAbs are not metabolized by CYP enzymes limits their toxicity and drug‐drug interactions. However, there are some exceptions, such as tocilizumab (Actemra), which induces the expression of CYP enzymes, increasing the clearance of other drugs metabolized by these enzymes.^46^ The main form of elimination for mAbs is cellular uptake by pinocytosis into the endosome, followed by intracellular metabolism through lysosomal degradation into peptides and amino acids.^13^ These catabolic products are then either used for protein synthesis or excreted by the kidney.^46^

The development of humanized and fully human mAbs has largely reduced immunogenic reactions to these therapeutic agents, but they are still possible.^46^ Potential adverse effects include those directly related to the target, for example, CD28 superagonist and the consequent cytokine storm,^50^ and immunogenicity, which is a risk factor that may be hard to predict or eliminate during development.^51^ Certain mAbs for immunological diseases can lead to immunodeficiency, leaving patients more susceptible to infectious diseases. For instance, due to the key role of TNF‐α in immunity to *Mycobacterium tuberculosis*, anti‐TNFs can lead to reactivation of latent tuberculosis.^52^ Chimeric antibodies are still on the market and can result in antidrug antibodies, leading to the loss of efficacy.^53^ Another consequence of the immunomodulatory properties of mAbs is their ability to cause autoimmune conditions, including lupus‐like syndromes, thyroid disease, and autoimmune colitis.^54^ Immunogenicity depends on both product‐related and patient‐related factors, and all of these should be considered during mAb development through an immunogenicity risk assessment.^55^ Nonimmune adverse events include a wide range of reactions, varying from headaches, mild gastrointestinal symptoms, and transient rashes to severe cytopenias; pulmonary, cardiac, hepatic, kidney and neurological toxicities. An example is abciximab (ReoPro), an antiplatelet antigen‐binding fragment that can produce acute thrombocytopenia following infusion.^54^

## 5 IMPROVING mAb ORAL BIOAVAILABILITY THROUGH FcRn: A CASE STUDY IN mAb PHARMACOLOGY

Monoclonal antibodies are not dosed orally because of the harsh pH conditions of the stomach, their cleavage and digestion by intestinal proteases, and their large size. Thus, mAbs are given by IV infusion on an outpatient basis, or by patient self‐administration using specialized SC injection pens or “needleless” devices. Adherence to systemically administered therapy has been scrutinized by systematic review and patient questionnaire analysis of diabetic patients on insulin therapy. Outpatient visits for infusions are inconvenient, and fear of injections or embarrassment of injecting in public is cited as reasons for nonadherence.^56^ Thus, there has been a long‐standing desire to provide patients an alternative in oral delivery of mAbs. A scenario could be envisioned where a high affinity and high efficacy mAb with long circulating half‐life could achieve therapeutic effect with acceptable cost of goods and dose after oral delivery.

It has been known for decades that receptor‐mediated IgG transcytosis is necessary for absorption of mAbs from the intestine, since permeation is limited to peptides of four amino acids or less.^57^ In addition to its role in IgG recycling and circulating half‐life (described above), FcRn is also present on intestinal epithelia.^58^ In neonates, IgG from maternal milk is taken up from the intestine into the circulation 59 allowing for passive immunity during the suckling period. In adult monkeys, which express intestinal FcRn, intragastric mAb dosing by endoscopy resulted in no detectable intact circulating mAbs.^60^ However, when mAb was dosed directly into the ileum of anesthetized monkeys, intact mAb was detected in circulation. Although the fractional uptake of mAbs was low (~0.3%), the time after dosing was extremely short (90 mins) and may not have allowed for complete absorption.^60^ These data and recent progress identifying the physiological conditions and mAb properties supported the following studies to attempt to achieve oral bioavailability in primates.

**Figure 1:**
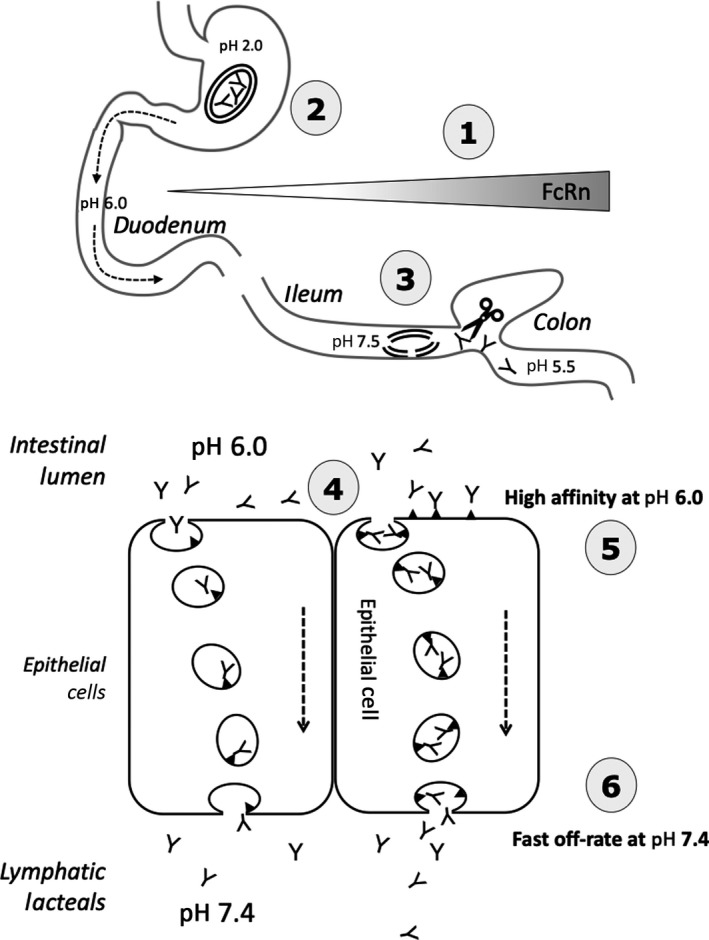
Working model used to establish the pharmacology of intestinal FcRn. This was used for the selection of a mAb in order to assess oral bioavailability in a 10 week dosing study in cynomolgus monkeys. 1. In human, FcRn expression increasing proximal‐distal gradient in the intestine. 2. Lyophilized mAb stable and loaded in sufficient amounts for dosing into enteric‐coated capsule protected from dissolution at low pH. 3. Enteric coating undergoes rapid dissolution at pH 7.5 in the terminal ileum to release mAbs that resist luminal proteases. 4. mAbs reach the apical surface of enterocytes and are limited by the rate of pinocytosis, unless there is IgG‐FcRn receptor surface binding. 5. Low pH favors mAbs binding at the apical cell surface or within the endosome, where they are trafficked to the basolateral side. 6. mAbs must have a fast off‐rate at pH 7.4 to reach lymphatic lacteals and eventually the systemic circulation. ▼, FcRn; Y, mAb; double lined oval, enteric coated capsule

The classic view of FcRn receptor‐mediated intestinal uptake is illustrated in Figure 1. The intestinal surrounding fluid is captured by pinocytosis on the apical surface of the enterocyte. Once IgG is internalized, the slightly acidic endosome (pH 6.0) favors the binding of membrane FcRn to soluble IgG. FcRn bound IgGs are trafficked to the cell surface, disassociate from FcRn in neutral pH 7.4 conditions, and are released extracellularly (Figure 1). Although FcRn binding affinity has been leveraged to extend mAb circulating half‐life,^48^ it had not been demonstrated for oral absorption in adult primates.

In suckling rat pups, FcRn expression and functionality is highest in the duodenum but disappears rapidly after weaning.^61^, ^62^ In contrast, in primate intestine, FcRn expression is highest distally in the intestine, in ileum/colon,^60^ and it persists in enterocytes through adulthood.^63^ To assess the feasibility of oral administration of mAbs, suckling rat pups were used to optimize FcRn‐binding affinity and pH conditions. Considerations, similar to small molecules, need to be taken into account when using tool mAbs in rodents. For example, mAb CDR binding to the rodent and human target protein should be comparable. This is analogous to the problem of speciation encountered by small molecules, especially those targeting GPCRs. If the human mAb CDR has reduced affinity to rodent receptors, then a separate tool mAb is generated, termed “surrogate” mAbs because they bind to the rodent receptor. Preclinical data provided by surrogate mAbs support the therapeutic efficacy and safety of targeting, but they are not the therapeutic molecule. In the case of FcRn, human IgG binds to rodent and human FcRn similarly in vitro.^64^ This was demonstrated in vivo by very active FcRn‐mediated human mAb uptake into serum after delivery directly into the small intestine. A nonengineered (wild‐type) mAb had fractional uptake up to 40% of the intestinal dose delivered.^62^ Pharmacokinetic studies established that mAb serum levels for oral uptake were a balance of FcRn affinity at pH 6.0 on the apical side and dissociation rate at pH 7.4 on the basolateral side into the interstitial fluid.^64^

In anesthetized adult cynomolgus monkeys, IgG1 mAbs dosed by direct infusion into the ileum showed much less fractional uptake than suckling rat pups. So, the question arose – was this because FcRn was not sufficiently active, or was it because of proteolysis known to occur in the hinge region of IgG1 mAbs. To prevent proteolysis in the hinge region, an alternative IgG isotype (IgG2) was generated to replace the IgG1 mAb used in rodents, while still retaining the same CDR sequence. After confirming that the IgG2 isotype had greater intestinal protease‐resistance than IgG1, as well as high FcRn binding affinity in vitro, the conditions for formulation and lyophilization were optimized. This was then loaded into enteric coated capsules designed to disintegrate and release the mAb in the ileum.^65^ This approach permitted repeat dosing over 10 weeks and facilitated any systemic accumulation due to the FcRn extended half‐life. Each capsule delivered approximately 3 mg/kg, and there was a doubling of the serum mAb level after the second dose, but this was not sustained, and further increasing the dosing still did not achieve serum levels above 1 ng/mL.^65^ Therefore, targeting FcRn expressed by enterocytes in adult primates to enable oral delivery of mAbs has been successfully demonstrated, but the efficiency of the process needs additional improvement before it can be considered for therapeutic application.

## 6 CONCLUSIONS AND FUTURE DIRECTIONS

Therapeutic mAbs have emerged as one of the fastest growing drug classes in history. Over the past few years, the market for mAbs has grown exponentially. Moreover, the mAb market is expected to continue to grow in the following years, given the large number of mAbs currently in development and the continued interest shown by pharmaceutical companies. These drugs can be developed for many indications, including cancer, autoimmune disorders and infectious diseases. Their highly desirable attributes, including high specificity, low toxicity, and immune‐modulatory activity, have paved the way for other engineered protein‐based biotechnology molecules, such as Fc‐fusion proteins, bispecific mAbs, ADC, and alternative scaffolds.

The introduction of humanized and fully human mAbs has helped to address the immunogenicity issues associated with the first‐generation rodent‐derived mAbs. Advances in the mAb field will depend on the ability to identify and validate novel targets and optimize the mAb structure to enhance the efficacy, optimize pharmacokinetic/pharmacodynamic properties, and minimize the potential side effects. The application of pharmacological principles, both in industry and academia, will increase proficiency in the development of better mAbs, especially for membrane‐bound targets. In parallel with this, other engineered molecules are being developed for the treatment of a wide range of diseases, which could not have been conceived of in the years before the development mAbs for therapeutic indications. To maximize both the therapeutic benefit and safety of this class of drugs, it is critical that their pharmacological properties be thoroughly characterized and understood.

## CONFLICT OF INTEREST

Dr Pamela Hornby is a full‐time employee of Janssen R&D. The rest of the authors declare no conflict of interest.

## AUTHOR CONTRIBUTIONS

Sofía Castelli is responsible for the content; Dr Hornby re‐organized to reduce the overall length and added a short section on FcRn pharmacology; Dr McGonigle edited and added insights that make it more meaningful for pharmacologists.

## References

[1] Cannon JG . Pharmacology for chemists, 2nd edn Oxford; New York: Oxford University Press; 2007.

[2] Kenakin TP . ProQuest (Firm): A pharmacology primer theory, applications, and methods, 2nd edn, Vol. xviii Burlington, MA; London: Academic Press; 2006:299.

[3] Leader B , Baca QJ , Golan DE . Protein therapeutics: a summary and pharmacological classification. Nat Rev Drug Discov. 2008;7:21‐39. 18097458 10.1038/nrd2399

[4] Levene AP , Singh G , Palmieri C . Therapeutic monoclonal antibodies in oncology. J R Soc Med. 2005;98:146‐152. 15805554 10.1258/jrsm.98.4.146 PMC1079437

[5] Kohler G , Milstein C . Derivation of specific antibody‐producing tissue culture and tumor lines by cell fusion. Eur J Immunol. 1976;6:511‐519. 825377 10.1002/eji.1830060713

[6] Breedveld FC . Therapeutic monoclonal antibodies. Lancet. 2000;355:735‐740. 10703815 10.1016/s0140-6736(00)01034-5

[7] Weiner LM . Fully human therapeutic monoclonal antibodies. J Immunother. 2006;29:1‐9. 16365595 10.1097/01.cji.0000192105.24583.83

[8] Yamashita M , Katakura Y , Shirahata S . Recent advances in the generation of human monoclonal antibody. Cytotechnology. 2007;55:55‐60. 19002994 10.1007/s10616-007-9072-5 PMC2104546

[9] Zhao A , Tohidkia MR , Siegel DL , Coukos G , Omidi Y . Phage antibody display libraries: a powerful antibody discovery platform for immunotherapy. Crit Rev Biotechnol. 2016;36:276‐289. 25394539 10.3109/07388551.2014.958978

[10] Strohl WR . Current progress in innovative engineered antibodies. Protein Cell. 2018;9:86‐120. 28822103 10.1007/s13238-017-0457-8 PMC5777977

[11] Kaplon H , Reichert JM . Antibodies to watch in 2019. MAbs. 2019;11:219‐238. 30516432 10.1080/19420862.2018.1556465 PMC6380461

[12] Mullard A . 2018 FDA drug approvals. Nat Rev Drug Discov. 2019;18:85‐89. 30710142 10.1038/d41573-019-00014-x

[13] Ryman JT , Meibohm B . Pharmacokinetics of monoclonal antibodies. CPT Pharmacometrics Syst Pharmacol. 2017;6:576‐588. 28653357 10.1002/psp4.12224 PMC5613179

[14] Geng X , Kong X , Hu H , et al. Research and development of therapeutic mAbs: an analysis based on pipeline projects. Hum Vaccin Immunother. 2015;11:2769‐2776. 26211701 10.1080/21645515.2015.1074362 PMC4916486

[15] Weiner LM , Surana R , Wang S . Monoclonal antibodies: versatile platforms for cancer immunotherapy. Nat Rev Immunol. 2010;10:317‐327. 20414205 10.1038/nri2744 PMC3508064

[16] Kinder M , Greenplate AR , Strohl WR , Jordan RE , Brezski RJ . An Fc engineering approach that modulates antibody‐dependent cytokine release without altering cell‐killing functions. MAbs. 2015;7:494‐504. 25933349 10.1080/19420862.2015.1022692 PMC4622058

[17] An Z , Forrest G , Moore R , et al. IgG2m4, an engineered antibody isotype with reduced Fc function. MAbs. 2009;1:572‐579. 20073128 10.4161/mabs.1.6.10185 PMC2791314

[18] Bruno V , Battaglia G , Nicoletti F . The advent of monoclonal antibodies in the treatment of chronic autoimmune diseases. Neurol Sci. 2011;31(Suppl 3):283‐288. 20644974 10.1007/s10072-010-0382-6

[19] Scott AM , Allison JP , Wolchok JD . Monoclonal antibodies in cancer therapy. Cancer Immun. 2012;12:14. 22896759 PMC3380347

[20] Davis TA , Czerwinski DK , Levy R . Therapy of B‐cell lymphoma with anti‐CD20 antibodies can result in the loss of CD20 antigen expression. Clin Cancer Res. 1999;5:611‐615. 10100713

[21] Lim SH , Beers SA , French RR , Johnson PW , Glennie MJ , Cragg MS . Anti‐CD20 monoclonal antibodies: historical and future perspectives. Haematologica. 2010;95:135‐143. 19773256 10.3324/haematol.2008.001628 PMC2805725

[22] Adams GP , Weiner LM . Monoclonal antibody therapy of cancer. Nat Biotechnol. 2005;23:1147‐1157. 16151408 10.1038/nbt1137

[23] Pento JT . Monoclonal antibodies for the treatment of cancer. Anticancer Res. 2017;37:5935‐5939. 29061772 10.21873/anticanres.12040

[24] Seidel JA , Otsuka A , Kabashima K . Anti‐PD‐1 and Anti‐CTLA‐4 therapies in cancer: mechanisms of action, efficacy, and limitations. Front Oncol. 2018;8:86. 29644214 10.3389/fonc.2018.00086 PMC5883082

[25] Topalian SL , Taube JM , Anders RA , Pardoll DM . Mechanism‐driven biomarkers to guide immune checkpoint blockade in cancer therapy. Nat Rev Cancer. 2016;16:275‐287. 27079802 10.1038/nrc.2016.36 PMC5381938

[26] Gong J , Chehrazi‐Raffle A , Reddi S , Salgia R . Development of PD‐1 and PD‐L1 inhibitors as a form of cancer immunotherapy: a comprehensive review of registration trials and future considerations. J Immunother Cancer. 2018;6:8. 29357948 10.1186/s40425-018-0316-z PMC5778665

[27] Dahan R , Barnhart BC , Li F , Yamniuk AP , Korman AJ , Ravetch JV . Therapeutic activity of agonistic, human anti‐CD40 monoclonal antibodies requires selective FcgammaR engagement. Cancer Cell. 2016;29:820‐831. 27265505 10.1016/j.ccell.2016.05.001 PMC4975533

[28] Flego M , Ascione A , Cianfriglia M , Vella S . Clinical development of monoclonal antibody‐based drugs in HIV and HCV diseases. BMC Med. 2013;11:4. 23289632 10.1186/1741-7015-11-4 PMC3565905

[29] Beccari MV , Mogle BT , Sidman EF , Mastro KA , Asiago‐Reddy E , Kufel WD . Ibalizumab, a novel monoclonal antibody for the management of multidrug‐resistant HIV‐1 infection. Antimicrob Agents Chemother. 2019;63:e00110‐e00119. 30885900 10.1128/AAC.00110-19 PMC6535568

[30] Song R , Franco D , Kao CY , Yu F , Huang Y , Ho DD . Epitope mapping of ibalizumab, a humanized anti‐CD4 monoclonal antibody with anti‐HIV‐1 activity in infected patients. J Virol. 2010;84:6935‐6942. 20463063 10.1128/JVI.00453-10 PMC2898252

[31] Sparrow E , Friede M , Sheikh M , Torvaldsen S . Therapeutic antibodies for infectious diseases. Bull World Health Organ. 2017;95:235‐237. 28250538 10.2471/BLT.16.178061 PMC5328111

[32] Yeung J , Holinstat M . Newer agents in antiplatelet therapy: a review. J Blood Med. 2012;3:33‐42. 22792011 10.2147/JBM.S25421 PMC3393068

[33] Levin M , Silberstein SD , Gilbert R , et al. Basic considerations for the use of monoclonal antibodies in migraine. Headache. 2018;58:1689‐1696. 30426478 10.1111/head.13439 PMC6283065

[34] Castle D , Robertson NP . Monoclonal antibodies for migraine: an update. J Neurol. 2018;265:1491‐1492. 29761294 10.1007/s00415-018-8886-8 PMC5990571

[35] Wang XY , Wang B , Wen YM . From therapeutic antibodies to immune complex vaccines. NPJ Vaccines. 2019;4:2. 30675393 10.1038/s41541-018-0095-z PMC6336872

[36] Cooper PR , Ciambrone GJ , Kliwinski CM , et al. Efflux of monoclonal antibodies from rat brain by neonatal Fc receptor, FcRn. Brain Res. 2013;1534:13‐21. 23978455 10.1016/j.brainres.2013.08.035

[37] Pardridge WM . Blood‐brain barrier drug delivery of IgG fusion proteins with a transferrin receptor monoclonal antibody. Expert Opin Drug Deliv. 2015;12:207‐222. 25138991 10.1517/17425247.2014.952627

[38] Karaoglu Hanzatian D , Schwartz A , Gizatullin F , et al. Brain uptake of multivalent and multi‐specific DVD‐Ig proteins after systemic administration. MAbs. 2018;10:765‐777. 29771629 10.1080/19420862.2018.1465159 PMC6150631

[39] Wang W , Soriano B , Chen Q . Glycan profiling of proteins using lectin binding by Surface Plasmon Resonance. Anal Biochem. 2017;538:53‐63. 28947169 10.1016/j.ab.2017.09.014

[40] Hinke SA , Cieniewicz AM , Kirchner T , et al. Unique pharmacology of a novel allosteric agonist/sensitizer insulin receptor monoclonal antibody. Mol Metab. 2018;10:87‐99. 29453154 10.1016/j.molmet.2018.01.014 PMC5985231

[41] Kang JC , Poovassery JS , Bansal P , et al. Engineering multivalent antibodies to target heregulin‐induced HER3 signaling in breast cancer cells. MAbs. 2014;6:340‐353. 24492289 10.4161/mabs.27658 PMC3984324

[42] Ovacik M , Lin K . Tutorial on monoclonal antibody pharmacokinetics and its considerations in early development. Clin Transl Sci. 2018;11:540‐552. 29877608 10.1111/cts.12567 PMC6226118

[43] Sedykh SE , Prinz VV , Buneva VN , Nevinsky GA . Bispecific antibodies: design, therapy, perspectives. Drug Des Devel Ther. 2018;12:195‐208. 10.2147/DDDT.S151282 PMC5784585 29403265

[44] Tsuchikama K , An Z . Antibody‐drug conjugates: recent advances in conjugation and linker chemistries. Protein Cell. 2018;9:33‐46. 27743348 10.1007/s13238-016-0323-0 PMC5777969

[45] Sau S , Alsaab HO , Kashaw SK , Tatiparti K , Iyer AK . Advances in antibody‐drug conjugates: A new era of targeted cancer therapy. Drug Discov Today. 2017;22:1547‐1556. 28627385 10.1016/j.drudis.2017.05.011 PMC6944323

[46] Keizer RJ , Huitema AD , Schellens JH , Beijnen JH . Clinical pharmacokinetics of therapeutic monoclonal antibodies. Clin Pharmacokinet. 2010;49:493‐507. 20608753 10.2165/11531280-000000000-00000

[47] Roopenian DC , Akilesh S . FcRn: the neonatal Fc receptor comes of age. Nat Rev Immunol. 2007;7:715‐725. 17703228 10.1038/nri2155

[48] Suzuki T , Ishii‐Watabe A , Tada M , et al. Importance of neonatal FcR in regulating the serum half‐life of therapeutic proteins containing the Fc domain of human IgG1: a comparative study of the affinity of monoclonal antibodies and Fc‐fusion proteins to human neonatal FcR. J Immunol. 2010;184:1968‐1976. 20083659 10.4049/jimmunol.0903296

[49] Chen X , Jiang X , Doddareddy R , et al. Development and translational application of a minimal physiologically based pharmacokinetic model for a monoclonal antibody against interleukin 23 (IL‐23) in IL‐23‐induced psoriasis‐like mice. J Pharmacol Exp Ther. 2018;365:140‐155. 29420255 10.1124/jpet.117.244855

[50] Bakacs T , Mehrishi JN , Moss RW . Ipilimumab (Yervoy) and the TGN1412 catastrophe. Immunobiology. 2012;217:583‐589. 21821307 10.1016/j.imbio.2011.07.005

[51] Singh SK . Impact of product‐related factors on immunogenicity of biotherapeutics. J Pharm Sci. 2011;100:354‐387. 20740683 10.1002/jps.22276

[52] Lorenzetti R , Zullo A , Ridola L , et al. Higher risk of tuberculosis reactivation when anti‐TNF is combined with immunosuppressive agents: a systematic review of randomized controlled trials. Ann Med. 2014;46:547‐554. 25105206 10.3109/07853890.2014.941919

[53] Baldo BA . Chimeric fusion proteins used for therapy: indications, mechanisms, and safety. Drug Saf. 2015;38:455‐479. 25832756 10.1007/s40264-015-0285-9

[54] Baldo BA . Safety of Biologics Therapy: Monoclonal Antibodies, Cytokines, Fusion Proteins, Hormones, Enzymes, Coagulation Proteins, Vaccines, Botulinum Toxins. Springer‐Verlag: SpringerLink (Online service); 2016. 10.1080/19420862.2017.1343709 PMC5540109 28678617

[55] Yin L , Chen X , Vicini P , Rup B , Hickling TP . Therapeutic outcomes, assessments, risk factors and mitigation efforts of immunogenicity of therapeutic protein products. Cell Immunol. 2015;295:118‐126. 25880103 10.1016/j.cellimm.2015.03.002

[56] Davies MJ , Gagliardino JJ , Gray LJ , Khunti K , Mohan V , Hughes R . Real‐world factors affecting adherence to insulin therapy in patients with Type 1 or Type 2 diabetes mellitus: a systematic review. Diabet Med. 2013;30:512‐524. 23323988 10.1111/dme.12128

[57] He YL , Murby S , Warhurst G , et al. Species differences in size discrimination in the paracellular pathway reflected by oral bioavailability of poly(ethylene glycol) and D‐peptides. J Pharm Sci. 1998;87:626‐633. 9572915 10.1021/js970120d

[58] Rodewald R , Kraehenbuhl JP . Receptor‐mediated transport of IgG. J Cell Biol. 1984;99(1 Pt 2):159s‐164s. 6235233 10.1083/jcb.99.1.159s PMC2275593

[59] Brambell FW . The transmission of immune globulins from the mother to the foetal and newborn young. Proc Nutr Soc. 1969;28:35‐41. 4182340

[60] Hornby PJ , Cooper PR , Kliwinski C , et al. Human and non‐human primate intestinal FcRn expression and immunoglobulin G transcytosis. Pharm Res. 2014;31:908‐922. 24072267 10.1007/s11095-013-1212-3 PMC3953555

[61] Martin MG , Wu SV , Walsh JH . Ontogenetic development and distribution of antibody transport and Fc receptor mRNA expression in rat intestine. Dig Dis Sci. 1997;42:1062‐1069. 9149063 10.1023/a:1018853506830

[62] Kliwinski C , Cooper PR , Perkinson R , et al. Contribution of FcRn binding to intestinal uptake of IgG in suckling rat pups and human FcRn‐transgenic mice. Am J Physiol Gastrointest Liver Physiol. 2013;304:G262‐G270. 23220220 10.1152/ajpgi.00340.2012

[63] Israel EJ , Taylor S , Wu Z , et al. Expression of the neonatal Fc receptor, FcRn, on human intestinal epithelial cells. Immunology. 1997;92:69‐74. 9370926 10.1046/j.1365-2567.1997.00326.x PMC1363983

[64] Cooper PR , Kliwinski CM , Perkinson RA , et al. The contribution of cell surface FcRn in monoclonal antibody serum uptake from the intestine in suckling rat pups. Front Pharmacol. 2014;5:225. 25339905 10.3389/fphar.2014.00225 PMC4188031

[65] Muzammil S , Mabus JR , Cooper PR , et al. FcRn binding is not sufficient for achieving systemic therapeutic levels of immunoglobulin G after oral delivery of enteric‐coated capsules in cynomolgus macaques. Pharmacol Res Perspect. 2016;4:e00218. 27433338 10.1002/prp2.218 PMC4876138

